# Supplementary material for: CRISPR/Cas9-Mediated Mutagenesis of Abdominal-A and Ultrabithorax in the Asian Corn Borer, Ostrinia furnacalis
Source: Insects. 2022 Apr 13;13(4):384. doi: 10.3390/insects13040384 (PMC9031573; doi:10.3390/insects13040384)
Supplement: Supplementary file 1 [file insects-13-00384-s001.zip › insects-1658593-supplementary.pdf]

Table S1. Primers used in this study.

Primer name	Primer sequence(5'-3')	Primer purpose
Abd-A-sgF1	TAATACGACTCACTATAGGGGTCGGCAAACTTACACG GTTTTAGAGCTAGAAATAGCAAGTTAAAATAAG	Preparation of sgRNA templates
Ubx-sgF1	TAATACGACTCACTATAGGTTATGGAGGGAAAGAATG GTTTTAGAGCTAGAAATAGCAAGTTAAAATAAG	Preparation of sgRNA templates
Ubx-sgF2	TAATACGACTCACTATAGGGTAGAAGGTGTGGTTGGT GTTTTAGAGCTAGAAATAGCAAGTTAAAATAAG	Preparation of sgRNA templates
GFP-sgF1	TAATACGACTCACTATAGGGCGAGGAGCTGTTCACCG GTTTTAGAGCTAGAAATAGCAAGTTAAAATAAG	Preparation of sgRNA templates
GFP-sgF2	TAATACGACTCACTATAGGCCACAAGTTCAGCGTGTC GTTTTAGAGCTAGAAATAGCAAGTTAAAATAAG	Preparation of sgRNA templates
sgRNA-R	AAAAGCACCGACTCGGTGCCACTTTTTCAAGTTGATAA CGGACTAGCCTTATTTTAACTTGCTATTTCT	Preparation of sgRNA templates
Abd-A-F1	TTCTCGCTGTTGAACTGCTT	Identification of somatic mutations
Abd-A-R1	TTACGTGGGGACTTTGTTCA	Identification of somatic mutations
Ubx-F1	ATGAACTCCTACTTTGAGCAGGGTG	Identification of somatic mutations
Ubx-R1	CTTATTGGGCGCTCTCTTACAAGAC	Identification of somatic mutations
Abd-A-qF	CGGCAAACTTACACGAGGTT	RT-qPCR for *Abdominal A*
Abd-A-qR	TCCTGCTCCTCTCTCTCTCG	gene (*Abd-A*)
Ubx -qF	CCACACCTTCTACCCTTGGA	RT-qPCR for *Ultrabithorax*
Ubx -qR	TCATCCTCCGATTCTGGAAC	gene (*Ubx*)
Lab-qF	CAACCAGCAGAACGGTTACA	RT-qPCR for *Labial* gene
Lab-qR	CCCCATAGTCCAGATTCGTG	(*Lab*)
Pb-qF	GGCTAAGCGGATCAAACAAG	RT-qPCR for *Proboscipedia*
Pb-qR	TGGGTAGTGACCGAGAGGAC	gene (*Pb*)
Dfd-qF	ATTACCATCCGTTGCCTCAG	RT-qPCR for *Deformed* (*Dfd*)
Dfd-qR	GGGCATGGAGTTTTCTACCA	gene
Scr-qF	TGGAGCACAAAGCTAACGTG	RT-qPCR *Sex combs reduced*
Scr-qR	CATTCGCATTCACCGTACTC	(*Scr*) gene
Antp-qF	AAGGATGCTCTGGACTCTGC	RT-qPCR for *Antennapedia*
Antp-qR	TATCTTCCGTCGCAGCTTCT	(*Antp*) gene
Abd-B-qF	AGTGGTGGCGAGTACAAACC	RT-qPCR for *Abdominal B*
Abd-B-qR	CTGACTCGGGAGCGTAGTTT	(*Abd-B*) gene
Wnt1-qF	ACGCCACACTGAGGAGAAAG	RT-qPCR for *Wingless*
Wnt1-qR	AACGCTGTCTCTCGACAACC	*Integrated family member 1* (*Wnt1*) gene
Dll-Qf	TATCCAACTCGGCAAATTCC	RT-qPCR for *Distal-less* (*Dll*)
Ddll-qR	GGAAGGGGTACCCTAACGAC	gene

Sca-qF	AGTACGTCCACGGATTTGGA	RT-qPCR for *Scabrous* (*Sca*) gene
Sca-qR	CTTGAAGCCACTCACGTTCA	
Transcript-qF	TTTATTGGCGATCCTGAACC	RT-qPCR for *Transcript* gene
Transcript-qR	GAATCCTCTCTGCCCAACTG	
Larp-qF	GCCTCTTCCGGTTCTACTCC	RT-qPCR for *La-related protein* (*Larp*) gene
Larp-qR	ACTTGTCGAGACACGCCTTT	
Centrosomin-qF	GCTTAGCACTTCCGGTAGGA	RT-qPCR for *Centrosomin* gene
Centrosomin-qR	ACATCTGCTTGTGGGAAAGG	
Dpp-qF	ACGGCTACGACGCCTACTAC	RT-qPCR for *decapentaplegic* (*Dpp*) gene
Dpp-qR	GCACCACATTGTTCACTTCG	
Connectin-qF	CCTTTTGGAAGGTGTTCGAG	RT-qPCR for *Connectin* gene
Connectin-qR	TCGCTTTTCGATTCGTTTCT	
Wnt4-qF	GCGTAGGCAGTTTCGGTAAG	RT-qPCR for *Wingless Integrated family member 4* (*Wnt4*) gene
Wnt4-qR	CGTGACATTTGCACACTTCC	
B-tubulin-qF	GCTGGTGGAGAACACAGACA	RT-qPCR for *Beta-tubulin* gene
B-tubulin -qR	ATGTTGACTGCGAGCTTCCT	
Spalt-qF	CACACCAAGGAACGACCTTT	RT-qPCR for *Spalt* gene
Spalt-qR	GTCAGCATGTGCTGCTTCAT	
Knot-qF	TTACACGGGGGACGATACAT	RT-qPCR for *Knot* gene
Knot-qR	TAGCCCGACATCAGACATGA	
Actin-qF	CCGTCCTCCTGACCGAGGCTC	RT-qPCR for *Actin* gene
Actin-qR	GGTGTGGGAGACACCATCTCCG	

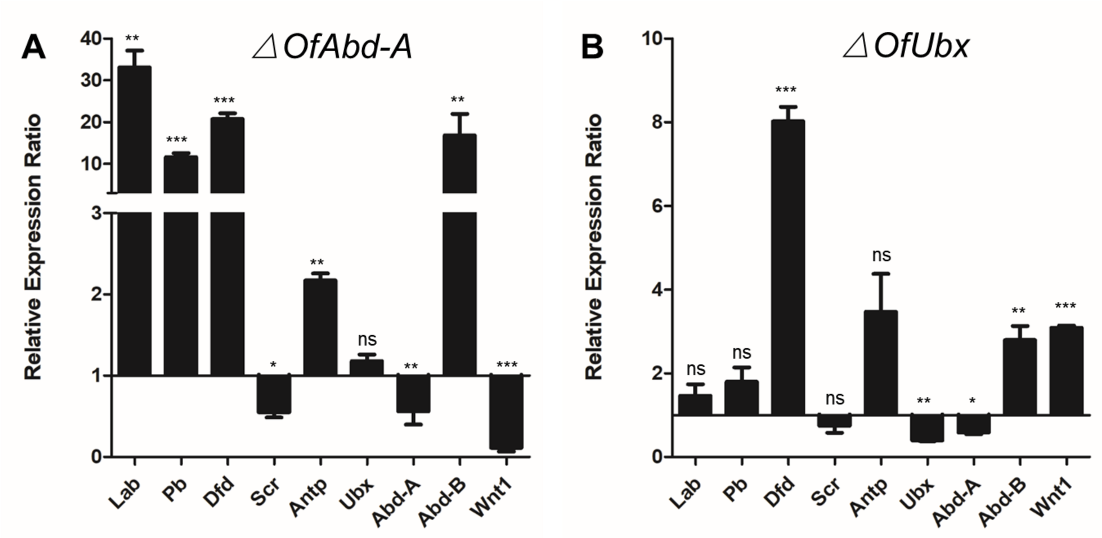

Figure S1. Relative transcript levels of downstream genes in *OfAbd-A* and *OfUbx* mutants.Transcript levels of indicated mRNAs in *OfAbd-A* (A) and *OfUbx* (B) mutants relative to wild-type levels. Three individual biological replicates of real-time PCR were performed. Asterisks (*,** or ***) indicate significant differences (P＜0.05, P＜

0.01 or P < 0.001, respectively) compared with wild type adults using a two-tailed t-test. ns represents no significant difference.

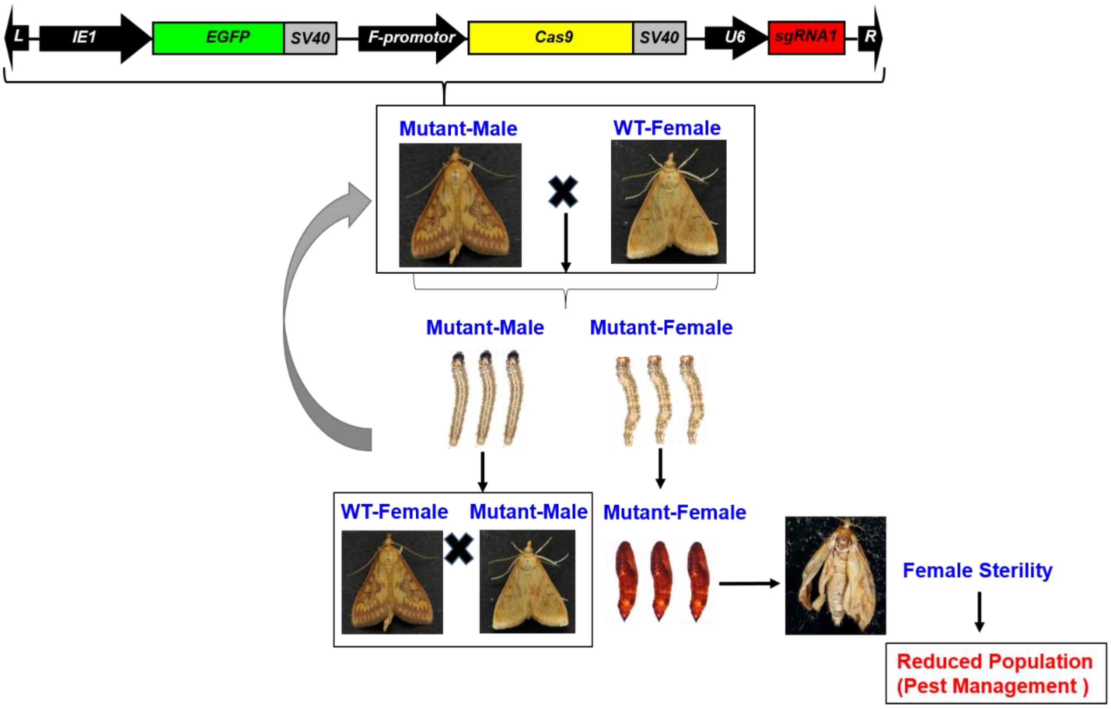

Figure S2. Prospective CRISPR/Cas9 mediated female specific transgenic pest control schematic.
